# Driving Into the Future

**DOI:** 10.3389/fpsyg.2020.574097

**Published:** 2020-09-18

**Authors:** P. A. Hancock

**Affiliations:** Department of Psychology and Institute of Simulation and Training, University of Central Florida, Orlando, FL, United States

**Keywords:** driving, automation, cognition, human factors, autonomous systems

## Abstract

This work considers the future of driving in terms of both its short- and long-term horizons. It conjectures that human-controlled driving will follow in the footsteps of a wide swath of other, now either residual or abandoned human occupations. Pursuits that have preceded it into oblivion. In this way, driving will dwindle down into only a few niche locales wherein enthusiasts will still persist, much in the way that steam train hobbyists now continue their own aspirational inclinations. Of course, the value of any such prognostication is in direct proportion to the degree that information is conveyed, and prospective uncertainty reduced. In more colloquial terms: the devil is in the details of these coming transitions. It is anticipated that we will see a progressive transformation of the composition of on-road traffic that will be registered most immediately in the realm of professional transportation in which the imperative for optimization exceeds that in virtually all other user segments. The transition from manual control to full automation will be more punctate than gradualist in its evolutionary development. As performance optimization slowly exhausts the commercial sector, it will progressively transition more into the discretionary realm by dint of simple technology transfer alone. The hedonic dimension of everyday driving will be dispersed and pursued by progressively fewer individuals. The traveling window of generational expectation will soon mean that human driving will be largely “forgotten,” as each sequential generation matures without this, still presently common experience. Indications of this stage of progress are beginning to be witnessed in the demographic profile of vehicle usage and ownership rates. The purpose of the exposition which follows is to consider and support each of these stated propositions.

## Introduction: A Short Glance Back – a Long Look Forward

There are many and varied forms of human work activities which have, across history, been undertaken. Each of these pursuits would have been considered commonplace, natural, and everyday actions to the contemporaries who witnessed them. In and amongst these, for example, the blacksmith and the peddler were, at one time, almost ubiquitous sights in the world. But now these particular activities, like many other occupations, have largely disappeared from the public landscape. And this to such an extent that we need to access our web-based search engines even to find out who “gas-lighters” and “hop-winders” actually were. These latter pursuits were both common enough and persisted well into the middle twentieth century. How much more recondite are occupations such as night-soilers, town-criers, fletchers, alewives, mudlarks, and gandy-dancers, to name only a few. Not many today could even say what these latter forms of work actually were, or to suggest how the product of these endeavors shaped everyday life at that time. We see that technology changes the functional landscape of work and inventions such as cell phones serve to exterminate jobs such as “tic-tac” man, even to the point that these jobs are now effectively forgotten. The nature of work changes and we, as individuals and society, change along with it (Hancock, 1997). However, none of the aforementioned pursuits, even in their own day, were ever as ubiquitous or as well-recognized, as that of “driver.” Indeed, drivers, in their many forms and incarnations (e.g., carters, teamsters, chauffeurs, truckers, pilots, steersmen, bicycle messengers, pyramid stone sled drivers, charioteers, etc.), have persisted now throughout an interval that can be even measured in multiples of millennia. As a result, our collective, social driving habits have been woven into the very fabric of civilized society and this driving enterprise is arguably an integral part of virtually all nominally “civilized” collectives. Few are the people who do not meet and encounter drivers regularly or indeed for that matter participate themselves in driving on a daily basis. But will drivers go the way of typesetters, switchboard-operators, or even more appositely, the human-computer; names which now ring only anachronistically and obscurely in our modern ears? Thus, the aspiration for the present evaluation is to consider and specify what precisely is driving us into the future? Given this ubiquity, the focus of the present work consists of an examination of the following important propositions. (i) What will compose driving in the future? (ii) With the onset of vehicle-control automation, will the profession and skill of driving fade, like others, into memory? and (iii) What of society in a world where no humans drive?

**FIGURE 1:**
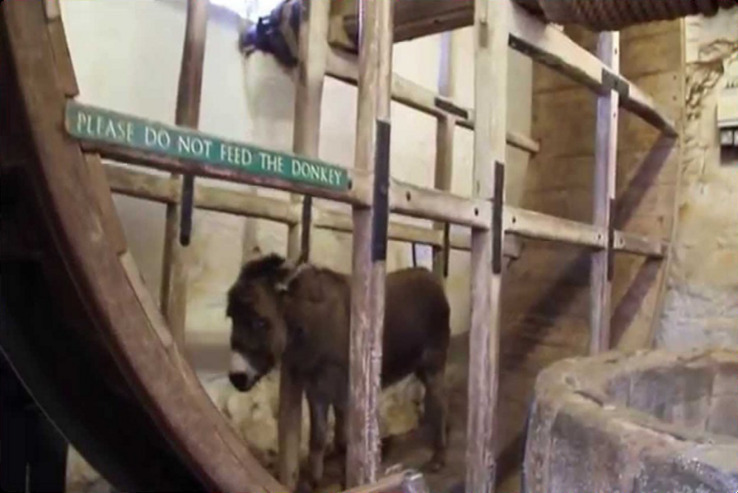
The donkey-powered water wheel of Carisbrooke Castle which was used to raise a water-bucket from an extremely deep well (at right). While the donkeys need “training” as to how to walk on the wheel, once that skill had been mastered, little in the way of subsequent human intervention is required.

As Yogi Berra is reputed to have observed, “*prediction is hard, especially about the future.”* However, the purpose here, as it has been in other associated works (e.g., Hancock, 2008), is to ruminate upon what, with respect to driving, is to come. The magnitude of change that promises to occur with the widespread penetration of autonomous vehicles (Rajasekhar and Jaswal, 2015), is very much in proportion to the extent of driving’s past and persistent longevity as well as its present ubiquity (and see Litman, 2017). Thus, the uncertainty which is associated with this anticipated degree of change is great in proportion (Hancock et al., 2019). To begin, we need first to briefly glance back in time in order to proceed cogently and thoughtfully into the future. Although it is not the purpose here to examine and rehearse the evolution of the specific role of driver in any exhaustive detail, it is enough to assert that we can find evidence of individuals in charge of some form of powered transportation back almost to the edges of recorded history. Mostly, this began by using animals as the power source, and with these capacities, drivers conveyed passengers and material (in all its forms), from origins to destinations; transport being the heart of trade and commerce and so the arteries and lifelines of civilization. Only consider here, for example the “Silk Road,” which covered even thousands of miles (Liu, 2010). The demand that was imposed upon early drivers tended, to some degree, to covary with the purported “intelligence” of the pack animals involved. Oxen proved to be sturdy but exhibited relatively little independent intentionality. Mules are hardy but, in human eyes, prove relatively stubborn compared to their close relative, the horse. Some animals, such as sled dogs, are viewed as exhibiting especially high levels of intrinsic intelligence, and so enabled the driver to proceed in a less immediately controlling manner. Regardless of the degree of these inherent levels of animal intelligence, if some rigid constraints could be imposed upon their actions, then selected animals can act almost independently (autonomously) from their human supervisor. If the constraining context is framed with sufficient ingenuity, e.g., a donkey on a wheel, then minimal human intervention may be required (see Figure 1). From this point of view, autonomous transportation is not necessarily a recent invention but one which, in differing guises, has been around for quite some time, e.g., “*Open Sesame*.” Perhaps the first watershed in driving, at least in terms of ground transportation, was when the source of power morphed from animal to artificial origins. The co-variation here with the Industrial Revolution being no happenstance. But now we stand on the verge of the next, and arguably, more profound watershed in vehicle functionality. This change is qualitatively and quantitatively distinct from any previous incarnation of transition. I frame the understanding of this approaching watershed in light of the recognized, and above referenced step-change, from animal to artificial power.

## From Living Muscle to Artificial Power

It is evident here that I have not featured any substantive discussion of wind-power and the comparable human conquest of the oceans as routes for trade and social interaction (e.g., Revelle, 1962; Campbell, 1995). However, in principle, each of the observations that I make are as applicable to “driving on the sea” as they are to “driving on the land.” To an extent, the nature of driving changed radically when artificially powered transportation became widely available. Effectively, the first of these forms of transport came about with the development of the steam-engine although, as I have noted, it can be argued that wind-powered vehicles preceded steam power, again even by millennia. In steam-powered trains, drivers tend largely to exercise control over one essential single degree of freedom (see e.g., Oborne et al., 2014), that being longitudinal velocity. Any degree of lateral control, i.e., route selection, point manipulation etc., tended to be the domain of another human decision-maker within the overall system. Although the train driver’s task consists of more than just controlling this one variable, the steam train footplate personnel were primarily involved in this activity either by facilitating or retarding longitudinal velocity on a momentary or more prolonged timescale. Some of these same strictures transferred to ground transportation when steam power was extended to on-road vehicles (Lay, 1992). However, it can be argued, and convincingly so, that horse-riders had already mastered many of these skills and the coachmen of the horse-drawn carriage era would transition fairly seamlessly into powered-vehicle chauffeurs, as history confirms. The inherent driving control skills themselves did not change radically with the transition of the source of power, but the growth of peak vehicle velocity began to impose higher, or more accurately different, cognitive demands upon the person at the controls.

It is not formally known, but can well be suggested, that the cognitive workload imposed upon early powered-vehicle drivers on the road proved to be an increment over that for say steam-train drivers. For, as we know, early steam-trains required a full footplate crew, whereas, effectively, steam-powered cars did not. The invention of the auto-starter also had important effects here since it obviated numerous procedural steps involved with vehicle activation, some of which required quite satisfying quite extensive degrees of physical workload. Here, the combination of physical and procedural constraints proved to be a barrier that technological advances could and did resolve (and see Möser, 2002). Also, we see that the roadway context of driving and the density of traffic began to exert further influences; although a horse-drawn carriage driver in downtown New York of the late nineteenth century would protest that their task had been no sinecure! This assertion about increments in workload can be a polemic one because each of these respectively identified roles were, and had been, composed of multiple tasks, as most professions were then and still are today (Hancock et al., 2020). Regardless of any such disputations, the evident fact is that each of the respective modes of transport were created or evolved so that human controllers could satisfactorily accomplish the task that was then set before them. There is little point in creating a vehicle that is absolutely uncontrollable. In sum, across the ages, driving has represented a satisficed not an optimized task (and see Simon, 1969), and the fabricated road environments vehicles occupy were specifically and intentionally structured to support this form of functionality and thus the associated level of sustainable cognitive demand. These various historical predicates for cognitive workload regulation mean that collisions have proved to be relatively infrequent. For example, the Insurance Institute for Highway Safety (IIHS), provide data that indicates that in the United States in 2018 there were only 1.13 fatalities per 100 million vehicle miles traveled (Insurance Institute for Highway Safety, 2020). This startling lack of fatal collisions becomes especially evident when we begin to consider the number of *potential opportunities* for collision on roadways, a point upon which I elaborate more below. The next threshold, which promises to be that of fully automated driving control is one decked with the “banners” of improvements in safety and efficiency (Kyriakidis et al., 2017; Hancock, 2018a; Hancock et al., 2020a). The empirical question, however, is whether autonomous vehicle collision avoidance capacities can now ubiquitously exceed the proven rates of human-mediated avoidance? In some sense, this question matches the evident degree of success of the human driver in coping with the imposed cognitive workload of driving vs. the equivalent capacity for autonomous vehicles to deal with that same imposed external demand. In the section which follows, I examine this proposition concerning the replacement of the human controller (driver). I aim to do this by throwing a purposive and explicit light on the quite remarkable abilities of humans to adapt to the satisficed demands of everyday driving. In short, my purpose, pro tem, is to emphasize just how good human beings actually are already at driving.

## The Most Practiced Skill in the World

**FIGURE 2:**
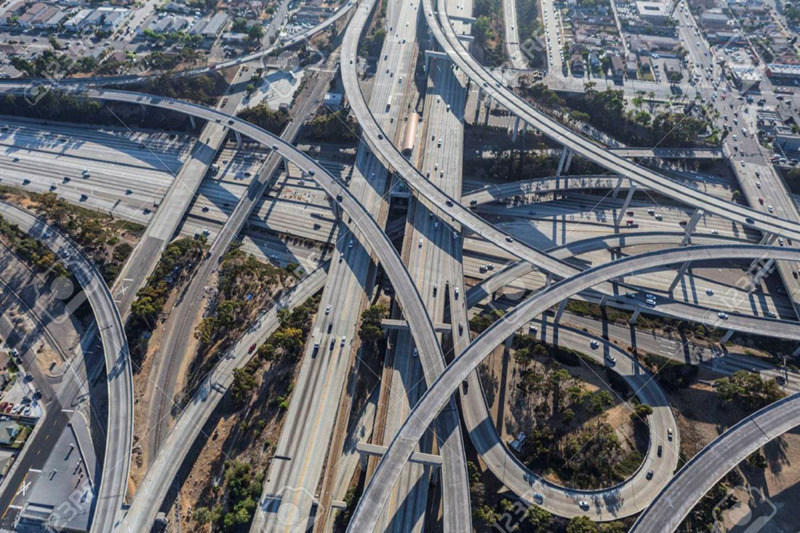
The nature of the urban landscape is massively influenced by the need to cater to powered vehicles and the requirements and ramifications of the overall transportation infrastructure (Ingram and Liu, 1999). It should also be noted that the nature of the rural landscape is also contingent upon this requirement, although materially, this can appear to be less impactful (Image reproduced with permission. iStock.com/Domepitpat).

Unlike many of the human professions and pursuits which, as we have seen, have now faded into the mists of time, driving has persisted and grown in proportion to the size of the populace and number of vehicles in circulation. To the present, we have experienced essentially two centuries of powered ground transportation with well over 100 years of individually driven automobiles. It is fair to say that the very landscape of countries such as the United States, Canada, and the like, have been sculpted by the presence and utility of the automobile and its particular needs (see Figure 2). In many, if not most cities of the world, the service of the automobile is a principal concern. Even in countries, such as those in Europe, where towns and cities were never explicitly or originally designed for the modern automobile, the car’s impact exerts considerable and persistent effects. This re-engineering of the cityscape to accommodate the automobile stands, to some degree, in concert with the way that the burgeoning sea-based trade of Amsterdam sculpted its reticulation of waterways and canals. Accommodations for vehicles either motivated or manipulated our modern urban landscapes and if we change vehicles’ functionality alongside the inevitable changes in the nature of the infrastructure that supports them, it promises to have a profound social impact, well beyond the confines of the vehicle itself (Stead and Vaddadi, 2019).

The area of urban design, the interaction with roadways and their influence is itself a research domain of vast extent (see e.g., Kenworthy, 2006). It is sufficient here to acknowledge these broad and diverse impacts. However, the present focus here is on individual human behavior in executing the role of driver. It is reasonable to assert that there is, and has been, no human skill more practiced than driving. True, all individuals do not engage in driving and so this is not a truly ubiquitous human practice (Hancock et al., 2009). Rather, the statement is applicable as more of a socially, nomothetic assessment. When we total up the number of commuters, professional drivers, and vehicle owners in general, it can be seen that vast swathes of the human race engage in this one activity and that it exceeds essentially any other singly practiced public skill; certainly in terms of the number of hours involved. It is also reasonable to assert that driving is the last great bastion of “analog” control. This does not mean that digital technologies are not involved in the control functions of modern vehicles, assuredly they are. Rather, it means that driving still requires the momentary exercise of psychomotor skill for continuous tracking whereas, in comparison, the vast majority of our other daily skills are now almost fully digital in nature and largely, or even solely require punctate mouse-clicks or button presses of the user. The exception here being video games which still feature this fulfilling, “tracking” aspect of human experience. As with many skills, and especially psychomotor skills, practice improves capability, even across periods of decades or more on the same task (Crossman, 1959).

With respect to such skill acquisition and its exhibition (Newell and Hancock, 1984; Hancock and Newell, 1985), humans are good at driving and are arguably, on average, even excellent at collision avoidance (cf., Yuris et al., 2019). But how can this be? We are all aware of the carnage on the roads and especially in the driving research community, we have been imbued with fatality and collision rates as the mantra of concern. But what we have never really attacked is the question of the relative rates of these human failures; that is, specification of the elusive denominator; the absolute number of non-events. In reality, how many non-collisions do actually occur per unit time to set against these adverse and life-altering vehicle accidents? It is quite natural for researchers, as well as the public in general, to focus upon the events that did occur and to direct scant, if any, residual attention to the events which did not. It is a human failing of both memory and ratiocination that we are poor at calibrating with respect to all forms of non-event. While there are clear evolutionary reasons why this neglect should be so, it still serves to bias our assessment in multiple areas, especially when the positive events prove as dramatic and life changing as a serious road collision. However, if we take as the unit of “non-collision” the space occupied by a vehicle multiplied by the time it occupies that space, and then reference this value to the frequency of actual collisions, which are represented by two vehicles or objects in transportation research (or more properly, any two material entities) occupying that same unit of space and time. If we were to conduct such a calculation, then human capacities must be well in excess of the 99.9999% reliability level. Arguably, it is even several orders of magnitude better than this. Of course, these levels of performance are not independent of increases in cognitive workload and effort, especially when driving conditions become demanding (Hancock et al., 1990). Automation does make it possible for people to begin to select the level of their participation, but they are not able to do so when automation in driving become obligatory. Nevertheless, this relative degree of human driver reliability makes the grand claims about safety gains for autonomous vehicles difficult to fulfill on both relative and absolute scales. The idea that eliminating driver error can be done by eliminating drivers is therefore rather problematic. As noted elsewhere, any absolute gains in collision reduction is a morally laudable achievement (Hancock, 2019a). However, the full scale of the issue in which both the numerator of collision frequency is juxtaposed to the denominator of non-collision frequency, has still to be even approximately identified, quantitatively speaking.

Claims for greater efficiency, in respect to transit times, may well suffer from similar lacunae in data specification. That is, individual transit times may be collected and plotted as putatively representative samples, but then their expansion and aggregation into assessment across the complete transportation system to hand is fraught with all of the perils of generalization. Obviously, as fully automated vehicles begin to predominate these associated electronic calculations become more tenable. However, in reality, the problem of mixed equipage, consisting of many automated vehicles interspersed with those of differing and nominally “lesser” capacity, serves to inhibit precise transit time specifications. However, the latter metric of change in transportation “efficiency” may perhaps, objectively, be somewhat easier to realize than the more safety critical failure-collision, non-collision ratio as has been mentioned above. Of course, these touted gains in efficiency are exactly what are held out as vitally supportive reasons for embracing automated driving in the first place. The mantra runs that automated vehicles can be “stacked” more efficiently together within the various roadways, traveling mere feet from each other in platoons, convoys, and the like. Technically, this has been shown to be a feasible configuration (see Lee et al., 2018). Yet what remains largely unproven are the outflow influences of these selective groupings on other traffic; presumably at some other juncture “embedded” within the same system. Tantalizing desiderata, such as greater “free time” is also offered to the innocent traveler, so inclined to adopt these commercially motivated innovations. However, as I have argued elsewhere, increasing “time” tends to be vacuumed up by the profit-driven system which then exploits the enhanced opportunity to have individuals now “work” on their way to work (Hancock, 2019c). In other words, this promise will largely serve to simply re-cast the physical location of, mostly, electronic work-based interactions. We have all become witness to this very phenomenon, as involved in response to the recent pandemic.

Critically, of course, lauding human driving abilities does not sell driverless vehicles. Nor does the defense of human capacities involved in driving greatly capture the attention of an information-jaded public. It seems, therefore, that the autonomous vehicle juggernaut will roll on despite any such observations (Daily et al., 2017). However, faced with this almost inevitable line of autonomy’s progress, the next consideration has to be one of the transition periods between human and computer control, and what challenges we are facing in the immediate, near-term. This particular challenge is tied to the presumed map of the transition phase bound up with the “levels of automation” taxonomy. This is most evidently articulated in the six Society of Automotive Engineers (SAE) levels conception to which I now turn.

## The Levels Autonomy Description and Its Associated Fallacies and Failures

To this point, the present work has been framed around the general arguments concerning the long arc of driving history. It will in its concluding section, proceed to some prognostications concerning driving’s future and some of the associated incarnation (and see also Pettersson and Karlsson, 2015). However, in terms of past, to present to future, it is important to consider the volatility and change embedded, especially within our own present transitional times (Hancock et al., 2013). The focus here is on the now, quite famous, and relatively ubiquitous “levels of automation” taxonomy and some pertinent critiques of it, as well as the path forward that it apparently offers. Criticisms here are somewhat palliated since the SAE construct does have facets of evident utility. Precisely where and how this formulation developed is a task for others to establish. Suffice it to say that I find that much of the thinking underlying these levels of automation formulation can be attributed to one of the luminaries of human-machine interaction; namely Thomas Sheridan (e.g., Sheridan and Verplank, 1978; Sheridan, 2002; and see Sheridan, 1992, p. 358, also Hancock and Sheridan, 2011). His *“ten levels of interaction”* proposition was one that could apply to many operational contexts and domains. Here, via the SAE promulgation, it has been directly applied to the transitional phases of driving control. There are a number of pertinent criticisms that are relevant to its application to the future of driving, whatever form that future driving takes (and see Parkes and Franzen, 1993).

**FIGURE 3:**
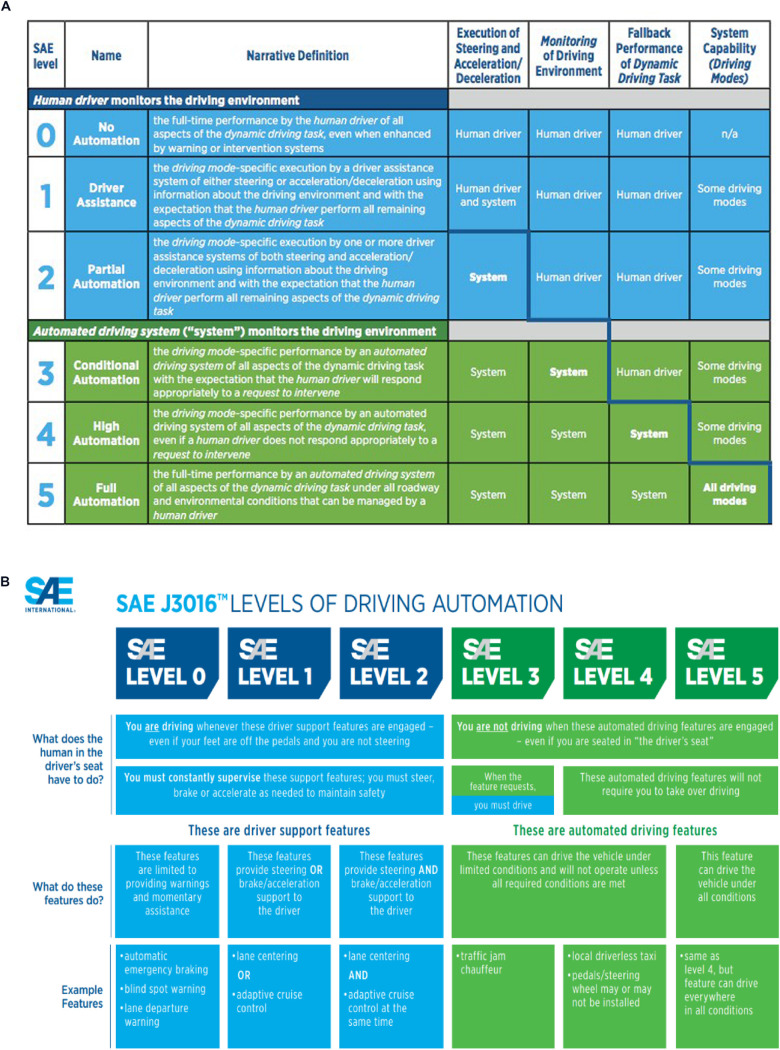
**(A,B)** SAE specified transition phases between no automation and full automation. The upper version is from 2014, the lower is the most recent revised version and is designated: SAE J3016 Standard: Levels of Driving Automation,” and is reproduced here with permission of SAE International. Propagated as a form of descriptive taxonomy, it promises to become a design ontology. Therein lies a number of debatable and potentially misleading assumptions as articulated in the text.

We can begin with the physical form of the SAE scale itself (see Figures 3A,B). The description begins at a zero level and provides what appears, putatively, to be a series of equal integer steps. The first impression is that we are looking at these respective steps from 0 and 5 on an apparent ratio scale, although whether this ratio implication was ever actually explicitly intended is probably rather doubtful. This implies an equal-interval structure between each of the discrete steps. This is a false implication and can be extremely misleading. It begins with the assertion that zero provides a no automation baseline state, but in itself this is also simply incorrect. Even for vehicles which well-preceded the modern, large-scale transportation thrusts such as the “*Intelligent Vehicle-Highway Systems”* (IVHS) (and see Hancock and Parasuraman, 1992), there was plenty of automation in cars, and some of it associated with immediate roadway control such as “cruise control.” This assumptive foundation of a zero level is evidently in error. A further implication of the SAE taxonomy is that each sequential step, between the respective levels, represents an equivalent change in functionality. As we are experiencing now, this implication is also false; most especially as we consider Stages 2 and 3 and compare them with Stage 4 for example. A further indication that this is an engineering-oriented perspective derives from the fact that there are actually six total levels identified, although the use of a zero anchor tends to convey otherwise. What this means is that there is no true intermediate step between the respective ends of what appears implicitly to be a continuum. The absence of such a “middle” state inhibits conceptualization to a degree that is not immediately obvious to users and/or designers who first encounter this influential representation. Some will chide that these objections as either rather trivial or only the musings of a nit-picking criticaster; but far from it. This representation has been taken as a form of *de facto* design “roadmap,” laying out apparently sequential and logical steps to achieve a fully autonomous future; a goal that itself is most often not adequately questioned. But it is the shortcomings in this conceptualization that presently threaten to lead to disruption and dysfunctionality. Of course, since the elaboration of transportation systems in the real-world is largely an empirical exploration anyway, and so no comparable “control” in order to assess the degree of any such dysfunctionality, is ever really feasible.

It is not simply the temptingly pristine representation of each individual step which proves to be problematic. The boundaries between each putative “level,” so readily and solidly illustrated in Figure 3A, and to a somewhat lesser extent in Figure 3B, are themselves frangible thresholds. In actuality, each sequential stage, at least to some degree, bleeds into some of its companions, and that not always to the level that is immediately adjacent to it (i.e., some elements of Stage 2 link directly to Stage 4, etc.). And underlying the whole illustration is the unstated, but highly influential implication, that “progress” necessarily requires us to move ever-upward in this hierarchy of levels (i.e., Stage 3 is two better than Stage 1, etc.). Through further subliminal suggestion, it also implies that full vehicle autonomy “must” be the socially desirable and ultimate design goal that we are aiming for. After all, in general is not anything better than zero? And if 5 is the top, is that not what we are aiming for? Rather than accept this assumption, it is one that we should most severely question (and see Hancock, 2014, 2017a). What precisely is the explicit, pragmatic need for automated vehicles? Do we not presently have enough humans on the face of the Earth in order to provide a sufficient number of controlling “drivers?” And when the evolving shibboleth of improved safety is once again paraded before a somewhat naive public and even trooped before professional scientists, let us ask expressly, where are the data to confirm this assertion? And most especially, where is the data that directly compares human safety records with automated control performance in exactly the same operational conditions? (and see Hancock, 2018a). Until this information is produced and analyzed, if indeed it is actually being recorded in such fully comparable instances, we will not know whether the whole process is indeed “safer,” or whether safety is once again being used as a “weasel” word to mean whatever its protagonists choose it to mean in order to convince others of their case (and see Hancock and Volante, 2020). There are no necessary reasons why many, if not all, of the assumptions embedded in the SAE levels description may not be flawed or simply incorrect.

With respect to the perspective promulgated in the five levels conception, there is another assumption, perhaps even more insidious for its unstated presence. This is that control is, in essence, a zero-sum function. In this concept, what any automated and autonomous systems gains, the human must necessarily lose. There have been numerous recent and cogent challenges to this assumption (see e.g., Shneiderman, 2020). There is no necessary reason why an overall expansion of mutual degrees of control could not be enacted i.e., a greater than zero-sum. This requires us to think of technologies more as team-mates than simple surrogates or direct replacements. The fallacy of the zero-sum of control persists only if we think in the constrained terms of monetary vehicle control. However, if we elevate our argument to a more macro-level concern for transportation and its diverse demands, this underlying restrictive premise is fractured, and the expansive vision of mutuality and sharing emerges (and see Gadsden and Habibi, 2009). In the various points discussed above, the “levels of automation” have, to a degree, morphed from an initial descriptive taxonomy to an eventual design ontology. As representative of our approaching transition into a new incarnation of driving, what is in essence the next watershed of driving itself, we need now to discuss the most immediate, problematic element of the SAE depiction as representing a form of an immediate future driving roadmap.

As has been discussed and elucidated elsewhere (e.g., Hancock, 2019a), one of the most pivotal issues of today concerns whether it is even feasible for a driver to recover full active control of their vehicle in the Stage 2 or Stage 3 conceptions of these proposed automation levels. While this might possibly be feasible in other realms of transportation (e.g., large container-ship control, and to a lesser extent commercial aircraft control; Scallen et al., 1996), the time-horizon limitations for successful resolution to momentary on-road challenges and/or automation failures seems sufficient to defeat the advocated and advertised resumption of manual control in these ground vehicles. It should also be noted that any innovative change, even in the other, arguably less temporally challenging transport circumstances (i.e., air, ocean), has been accompanied by transient increases in failure rates. We must be prepared for these spikes in adverse outcomes, as is discussed in more detail below (and see Hancock, 2011, 2019a). Most especially, this seems to apply to vehicles in high-density traffic situations, and/or on high-velocity roadways. It is not that we cannot conceive of these control transitioning technologies, but rather whether it is practicable, feasible, and even advisable to pursue these forms of control return strategy (Desmond et al., 1998). For, in these respective stages, the “driver’s” role is translated into one of passive monitoring, which is a role that we understand already that humans can be extremely poor at (Hancock and Warm, 1989; Hancock, 2013b, 2017b). Response latencies increase across time in such vigilance situations (Hancock, 2017b), as do the frequency of missed signals as epitomized in the well-recognized “*vigilance decrement function*.”

Although Stages 2 and 3 are more than difficult to manage in terms of human-vehicle interfaces and recovery response, if we do choose to adopt these forms of transition, it may be especially pertinent to consider a “driver command by negation” architecture (and see Hancock and Verwey, 1997). Here, the automation temporarily assumes complete command and so communicates that state through differing perceptual modes (e.g., voice warnings, visual icons, etc.). The only response required of the driver at this juncture is a no statement. This form of interaction does not require any form of an affirmation, i.e., “I agree.” Rather, the human requirement here is only an interruptive “command by negation,” i.e., “I disagree.” Pilot command by negation (PCN) is quite a well-known as a human-machine communication strategy, especially in aviation (Hancock, 2007). This command structure follows an old maxim in law being *qui tacet consentit* or, “he who is silent agrees,” or even more simply, “silence gives consent.” However, it is almost without doubt that the time constants involved in ground-vehicle control takeovers will eventually defeat any form of human interaction after putatively regaining such control (Hancock, 2019a). In terms of driving, the temporal paradox might be expressed as follows. The Stage 2 and 3 takeover policies almost inevitably require that we know the future beyond the time horizon that normative perception-response provides. That being so, we would need a degree of prospection that we do not currently possess. Indeed, if we did possess prospection to this degree, we would already be able to anticipate that future event successfully enough that take-over would be unnecessary. The temporal constraints of the human response system itself prevents this from happening (Hancock and Weaver, 2005; Francis et al., 2020).

One further unwritten sub-text here is one that features the issues of control and legal liability. For, if we employ the traditional vision of liability, then the individual human driver is both responsible for control and thus at fault when failure occurs. However, if we employ a systems-based perspective, the skein of responsibility becomes much more complicated and potentially exposes the vehicle’s manufacturers, and their constituent sub-contractors, in a way inimical to their own best interests. This is one of the touchpoints where the radical differing “magesteria” of technology (science) meets that of the law (Hancock, 2020). It will be especially edifying to witness how the two competing visions of driving, i.e., the commitment to driverless vehicles vs. the shared control/driver assist strategies play out in the coming decade, especially in light of this liability issue. Given the tide of technological progress, it appears the long-term winner is basically already decided in the ground transport realm. However, the race is still in progress and the forces which continue to favor a human driver-centered approach are by no means negligible.

A final issue upon which we can reflect concerning human interaction with autonomous vehicles is the concern for attribution error (Hancock et al., 2020b; Stanton et al., 2020). Much of the interaction on our roadways is mediated currently by implicit communication between human drivers. Such behaviors are, for example, evident at unregulated intersections, where eye-contact can mediate arrival and departure priority. Attribution is also contingent upon facets of behavior such as courtesy and etiquette. These self-same strictures are in action when drivers interact with other road users such as pedestrians, bicyclists, etc. Here, shared common assumptions and expectations mean that it is not only the formal rules of the road which guide action but informal, social ones also. This is also why driving in differing countries with different cultures and varying implicit assumptions can prove to be rather stressful. The central point here being that courtesy, empathy, and implicit knowledge are not yet built into automated vehicles nor their controlling software. Nor does there appear to be any great customer clamor for manufacturers to do so. The question that we can ask is whether such attributional dissonance between human and automated vehicle will necessarily lead to conflict, confusion, and collision (Hancock, 2018b). This concern is, of course, one that is greater than automated terrestrial vehicles, for it asks questions about our physical interaction with all other objects, and most especially advanced forms of technology. For example, how are robots supposed to react to the presence and motion of people, both their users and others in their ambient environment? Here, the principles of biomimesis gives us a lead. For, in nature, we often implicitly understand our role in gatherings such as crowds, or in unusual situations such as cattle stampedes or during the running of the bulls (in the latter situations, getting out of the path of the animals being a recommended strategy). These sorts of principles of self-organization and self-separation are now being codified into advanced commercial aircraft. It is almost certain that they will be incorporated into driverless vehicles also. In conclusion, with respect to the present stage of control transition, the SAE description has proved to be a provocative and probing proposition. While not without some element of value, it has served to both frame and constrain the avenues of progress in the driverless vehicle world to arguably, a disproportionate degree of influence.

Although not engaging in prescriptive designations *per se*, it still remains possible to consider the relative advantages and disadvantages of both human-centered and automation-focused driving propositions, and this I have presented in Table 1 which follows. I am very aware of the propensity to couch these forms of observation in the comparative terms of so-called MABA-MABA (men are better at; machines are better at) types of juxtaposition. Indeed, the merits of such contrasts have previously been discussed and debated, and that rather extensively so (see e.g., Sheridan et al., 1998; Dekker and Woods, 2002; Hancock, 2007; De Winter and Hancock, 2015). As a result, the observations given in Table 1 serve more as points of discussion for greater scientific and social consideration rather than hard and fast rules for function allocation between either human, automation, or human and automation together working in some form of concert.

**TABLE 1:** Side-by-side descriptions of a series of advantages and disadvantages for human control juxtaposed with automated control.

Driver-controlled		Automation-controlled	

Advantages	Disadvantages	Advantages	Disadvantages
Significant pool of accumulated skill	Obligatory task focus for effective Performance	Advertised superiority in transit efficiency	Potential violation of personal privacy
Often able to respond to unexpected events	Human controller suffers from progressive fatigue	Advertised reduction in collision frequency/intensity	Vulnerable to cyber attack
Proven low relative error rate	Vulnerable to stress and workload disruptions	Readily scalable for widespread utilization	Difficulty in dealing with bespoken challenges
Control fosters individual self-efficacy	Poor at extensive monitoring activity	Ready inter-operability with other automated systems	Contemporary lack of all affect
Capable of subtle forms of pattern-recognition	Relatively slow rates of skill accumulation	Little performance degradation across time	Imposes constraints on personal human freedom
Uses most complex control mechanism currently known	At risk for distraction during active control	On-line, remote operational improvements possible	Non-transparent operational states
Able to experience joy and fulfillment	Large individual differences across user population	Currently perceived as “inevitable.”	Vulnerable to rider distrust and neglect of use

*The vulnerabilities of this structure to misinterpretation as deterministic compositions between the relative capacities of human and machine are articulated in the associated text. Descriptions of the team advantages and disadvantages have not been presented in the Table but are discussed in the text.*

In general, we have become well aware of the capabilities and flaws associated with human behavior (e.g., see Hancock and Matthews, 2015). In particular, drivers can become fatigued, stressed, and/or distracted (Hancock et al., 2003). In part reaction, the promulgated advocacy for automation is that automated vehicles would not be vulnerable to these influences. Similarly, human drivers can only tolerate certain absolute levels of task-related workload, and theories of driving have even been predicated upon each individual’s management of this, their own dynamic levels of regulated task demand (see e.g., Fuller, 2005). Further, although human drivers can, in general, satisfy the demands placed upon them, there remain large differences in individual capacity which means a lack of uniformity of competence of the human drivers on our roadways.

Yet the promised alleviation of these concerns for human shortfalls by aspiringly autonomous vehicles does not itself come without costs. Members of the driving community, in giving up control, also give up some degree of personal privacy. They certainly give up some aspects of personal autonomy and in so doing they must interact with machines whose full spectrum of functions they need not necessarily either understand or trust (see Hancock et al., 2011a, b). Such machines are, like all computer systems, to a degree, vulnerable to cyber-attack. Rational decisions as to the strategy adopted for future transportation ought to be founded on factually supported assessments of the veracity of purported gains of automation, as compared to what is frequently advertised by those wishing to sell advanced vehicles (Hancock et al., 2019). In respect of both the promised gains in safety, in terms of reduced frequency of collision, and in terms of gains in trip time efficiency, the data should be determinative over advocational publicity. Sadly, as with many such social policies, this does not promise to be so.

Many of the present concerns with the approaching incursion of automated vehicles are ones that are necessarily embedded in the period of transition that we currently inhabit. It is also important to note that driving is only one example of this transition, as the same issues task many other operational domains. This general debate is articulated in further detail in the section which immediately follows. In a number of ways, this global transition from human to computer is epitomized in the description of SAE Stages 2–4 in which the emigration of control from the human is taken up, in a form of zero-sum way, by the attendant automation. However, as noted earlier, progress through each of the discrete stages is neither necessary nor obligatory. However, as with comparable innovations in advanced aviation operations, we will witness various transient effects during this epoch of transition. These transients have to be recognized so that collectively, we can be cognizant of the degree that the outcome pattern of performance witnessed is reflective of the novel elements associated with such transitions, as opposed to any fundamental flaws in any particular line of technical development. The latter concern emerges when failure rates spike and companies and institutions, whose personnel are attuned to such acute changes in event numbers, tend to react accordingly. Much here depends upon the business cases made for the innovations offered and the way that the market responds to these technological offerings. There is also, of course, an overall propensity to reject retrenchment such that when a new system is initiated, it proves hard if not impossible to return to the older approach, should the new one fail to deliver on its advertised promises. This form of antipathy to putatively “backward” steps in technology is a powerful force, and one to be reckoned with.

In ground transportation, the period of transition is liable to be quite a prolonged one. More formally, the percentage prevalence of on-road legacy systems is liable to be high. This is in part because, like the function of transportation itself, vehicles are not exclusively utilitarian in nature. Indeed, a non-trivial proportion of travel is undertaken for purely hedonic reasons and the ownership of vehicles is not solely for pragmatic transit, but often for the pleasure of ownership. Like other high legacy systems, such as firearms, there will then persist in use a broad mixture of age and capability of on-road vehicles. The efforts of infrastructure designers to parse these various segments of the traveling inventory into differing regions of spatial operations, or temporal distinctions in terms of permitted hours of operation, will be motivated by the imperatives of efficiency. However, these forces will be pitted against the persistent desire for freedom of operation. It is liable to be a complex and polemic trade-off involving such opposing forces. And across this checkered landscape is emerging the observed trend for reduction in vehicle ownership rates, especially among the cadre of younger drivers (cf., Shaheen and Cohen, 2013; Knittel and Murphy, 2019). As to the stability of these trends and their social, cultural, and national variations across the globe, the trends are rather regionally contingent. However, there is little doubt that the injection of permanent circulating autonomous vehicles for hire and their immediate availability via smartphone linkages will further serve to influence vehicle purchase decisions as future generations face their own mobility challenges. In sum, transition is liable to be a motif of transport systems for some time yet to come. However, transport exerts wider influences than simple passage between origin and destination alone. It is to these impacts that I next turn.

## Driving as the “Wedge” Issue: To Change Driving Is to Change Society

In many ways, driving very much represents the “thick end of the wedge.” It is, what I have thus termed the modal, “wedge” issue concerning the penetration of ever more autonomous systems into human life (Hancock, 2015, 2019b). In the steps from an analog to a digital world, driving retains at least the vestiges of a past and passing era. Lives across the world have already been vastly transformed by this revolution, but now the tide of such change is attacking perhaps its last and most formidable bastion. As such, we are not simply looking at the future of driving, we are surveying what promises to be a differing way of social organization and human existence. The issue of momentary control is embedded within a much wider concern for personal autonomy. That is, when we relinquish the effective momentary control of the vehicle, we are also abdicating from the full expression of freedom that it represents. Future vehicles will come pre-programmed with multiple origins and destinations; and even some present vehicles have these resident capacities. Departures from these everyday journeys linking home, work, grocery store etc., will be exception processed. But just as no man is an island, so no autonomous vehicle will actually be completely autonomous. That is, such vehicles will necessarily be embedded into a systems-wide integrated transport system that will require to know about each componential element; where it is coming from and where it is going to, and when. While this requirement might well make it harder to accomplish “getaways” after robberies, a constraint that socially we might approve of, it will also interfere with many other more provocative dimensions of privacy. For example, how do we organize a surprise party, when the data are necessarily available to let inquirers know exactly where one’s friends and colleagues are? This might be a fairly puerile example, but it does serve to make the point; there are occasions upon which people do not wish to let anyone or anything know where they are going. Changing the nature of momentary vehicle control thus has outflows into society that need be neither immediately obvious nor easily anticipatable. As mentioned in the summary here, we also neglect to consider the pleasures of driving at our peril.

As the proponents and pilgrims of higher, automated “safety” wend their unhindered ways through the lines of social discourse, what of those who still personally want to drive? Must their pursuit be limited to out of the way facilities, fit only for enthusiastic hobbyists? Will we not lose something more than a symbol when the steering wheel is finally abandoned? Driving is not merely the simple act of vehicle control; it is a declaration of personal expression. In a world where the natural propensity of the dominant consumerist system is to curtail such elaborative forms of human behavior, automated control of one’s personal vehicle represents another step along the straight-jacketed road to obligatory social conformity. Let us then beware of what is here driving us into the future. For, it may not be the panoramic promise of autopia that we are being taught to visualize, but something potentially much more disturbing.

## Are We There Yet?

How far off is the future? This is always a thorny conundrum. No one disputes the fact that semi-automated vehicles have already been “let loose” in the world to conduct an informal empirical exploration of their capacities in the wild as it were. And these more recent incarnations are inevitably introduced into worlds which, for the foreseeable future will, as has been noted, still contain various and traditional forms of human vehicle control. With temporary transference of human driver skills across vehicles, such as that experienced in renting a car, it may be that drivers will traverse the differing levels of tactical and strategic control, as represented by Stages 2 and 4 of the SAE specification. This might even be envisaged within truly short periods of time, such as the transfer that occurs when one rents a totally different model and generation of vehicle. In this general sense the introduction of more automated vehicles, is no different from offering new iPhones, Tablets, and other advanced forms of computational consumer systems which are designed but never exhaustively tested before deployment. The issue here is that these new technologies control a one-ton vehicle proceeding at 60 mph and glitches, faults, bugs, and errors are not merely frustrating, they can be fatal (and see Templeton, 2020). But is this same process not as true for other, equally safety-critical systems such as advanced fly-by-wire aircraft whose similar failure we have witnessed in recent months? It might be suspected that in rather the same way that an aircraft crash draws widespread news coverage and single vehicle fatalities much less public attention, so the respective flaws in automated ground transportation will draw neither the same level of social disapprobation, nor the same level of regulatory scrutiny as is visited upon commercial aircraft and their functioning. We might well hope this is not the case, but precedent militates against this aspiration. Here, again, time will tell the tale.

At one and the same time that we are about to radically change the nature of physical transportation and its control, we are witnessing a forced proliferation of the “electronic” transportation of information. Here data is, to the greatest degree possible, substituted for material, and the transport of data is so much more easily achieved. Why “port” material goods if, for example, 3-D printers can take remote instruction and create a need item on the spot? The pandemic, which began essentially at the start of 2020, now engulfing our world has forced a re-casting of movement imperatives. Here, much discretionary, and elective travel has been curtailed, or at least stultified (and see Ellwood, 2020). Ways are now being envisaged to extend that propensity to what has previously been viewed as obligatory transportation when, assumedly, the pandemic subsides (cf., Freedman, 2017). At the same time, the whole demographic of the driving public is itself in a state of flux. Today’s younger generation no longer see vehicle ownership as obligatory or even perhaps even preferable. Services such as Uber, Lyft, etc., tend to mean that personal travel is an on-demand requirement that does not necessarily entail a vehicle of one’s own. Timeshared vehicles and on-demand transport are even more likely to burgeon if pure driverless (and perhaps individually ownerless) vehicles circulate in local environments. If a number of these trends are sustained, and if they are underwritten by greater profit, and there is no reason to believe they will not be, then motivations for goals such as improved roadway capacity may begin to dissipate as the absolute level of vehicle numbers on roadways are themselves reduced. This is another of the promises held out to the public to persuade them toward collective acceptance.

What were once thought to be rather fanciful notions, e.g., the delivery of small packages by purpose-directed drones, now look much more realistic in light of the times in which we live. Rather paradoxically, the collision rates, which assumedly ought, at least to a degree co-vary with the number of vehicles on the road (Hancock, 2013a) does not seem to follow any such simple relationship. Such patterns challenge us to understand what safety levels will be like in an increasingly mixed equipage situation (Sivak and Schoettle, 2015). There might, for example, be the opportunity for low-level flying personal transport to be substituted for on-road vehicles. All this is to say that we are now fully engulfed in perhaps the next great wave of powered transportations evolution. If the first wave was characterized by the replacement of human muscle with animal power, and the second the replacement of animal strength by artificial power, then the third wave is most certainly the one where human movement control is abrogated to a computational surrogate. As each of these steps were accompanied by fairly radical changes in the organization and structure of human society, so we cannot but expect that the latter will exert both anticipated, but also unknown effects in a similar manner. Driving is not merely the act of vehicle control; it is a relationship between a person and the world in which they live (Singer, 2014). For good or bad, we are changing driving’s rules and with them the very role of vehicles in society itself (Howey, 2012). The step-change that we are in the middle of will recast the world as we understand it.

## A Summary Not Yet Concluded

As we conclude with a vision of a slowly diminishing, then dying, then virtually an extinct activity of driving, we must spare a final thought for those who actually love it as a desired pursuit. Here, we not only include those that actually rely on driving for their profession, such as taxi drivers, truck drivers and the like, but also for those various ancillary enthusiasts. What of those whose profession is a Formula One driver, NASCAR racer, dragster, and those who enjoy watching these forms of vehicle-related entertainment? Will crowds turn out to watch autonomous stock car racing in the same way we now have a niche for “battling” robots? Perhaps, but one senses not. How will Formula One look when an fully designed, tested, and evaluated optimal control algorithm can easily exceed even the greatest human exponent of the art? And what of the simple, plain experience of satisfaction in controlling one’s own destiny on the open highway? Will books such as “*Zen and the Art of Motorcycle Maintenance*” (Pirsig, 1974) be inspired by autonomous motorcycles? There is therefore an important hedonomic dimension to driving (Hancock et al., 2005; Hagman, 2010) which extends well beyond the mere utilitarian necessity to relocate persons and material from origin to destination. Of course, these pleasurable and hedonic values have to be set against the traditional concerns in driving for downsides such as pollution, driving’s subsequent contribution to global warming, the problems of over-crowding and time-wasting in queues and gridlock etc. However, if driving is the wedge issue that I have identified, then it is possible to postulate that many presently gratifying, associated human activities will be submerged and then extirpated by the insensible tide of spreading autonomy. It will not be too long before our children may ask, in all naivete: “what was a driver?” And after that what? The changes to society will extend well beyond only these vanishing hedonomic dimensions. Most disturbingly, the growth of independent, autonomous vehicles will, without careful political legislation, serve very much to limit human freedom. In the same way that even presently existing video surveillance systems curtail and constrain people’s actions, the inability to “*see the USA in your Chevrolet*,” is something more than just transferring momentary vehicle control from a human to an AI-based autonomous substitute. Rather, it promises to represent a profound change in the human condition. When driving into the future, we need to be much more wary about the coming roads, whether they be ones either more or less traveled.

## Author Contributions

The author confirms being the sole contributor of this work and has approved it for publication.

## Conflict of Interest

The author declares that the research was conducted in the absence of any commercial or financial relationships that could be construed as a potential conflict of interest.
